# Pyroptosis-Related Gene Signature and Expression Patterns in the Deterioration of Atherosclerosis

**DOI:** 10.1155/2022/1356618

**Published:** 2022-05-05

**Authors:** Yan Wu, Qi Ma, Xudong Wang, Tianci Wei, Jiawei Tian, Wenjing Zhang

**Affiliations:** ^1^Department of Ultrasound, The Second Affiliated Hospital of Harbin Medical University, Harbin, China; ^2^Department of Ultrasound, Harbin Red Cross Central Hospital, Harbin, China; ^3^Department of Laboratory Diagnosis, The Second Affiliated Hospital of Harbin Medical University, Harbin, China

## Abstract

**Background:**

Pyroptosis has been shown to be involved in the overall process of atherosclerosis. This study was aimed at investigating pyroptosis-related gene expression patterns in atherosclerosis and their diagnostic significance.

**Methods and Results:**

In GSE100927, fifty-four pyroptosis-related genes were identified. Between atherosclerotic plaques and normal samples, the expression patterns of pyroptosis-related genes were significantly different. In order to construct a pyroptosis-related risk score signature (PRSS), the least absolute shrinkage and selection operator (LASSO) was combined with multivariate logistic regression to screen twelve genes. The diagnostic efficiency of the PRSS performed well in GSE43292, as shown by the results of receiver-operating characteristics (ROCs). Consensus clustering identified two expression patterns of pyroptosis-related genes in different statuses of atherosclerotic plaque in GSE163154. The biological behavior of the different clusters was examined by the gene set variation analysis (GSVA). The Kyoto Encyclopedia of Genes and Genomes (KEGG) and Gene Ontology (GO) enrichment analyses revealed that the differentially expressed genes (DEGs) in the two clusters were enriched in the immune response. The Cytoscape software was used to construct protein-protein interaction (PPI) networks for hub gene screening. Following that, the Drug Gene Interaction Database (DGIdb) was utilized to find 47 possible medicines and chemical compounds that interact with hub genes in atherosclerotic plaques.

**Conclusion:**

The results of this study showed that pyroptosis-related genes contribute to the progression of atherosclerosis and may serve as biomarkers in clinical diagnosis as well as novel therapeutic targets for the treatment of AS.

## 1. Introduction

Atherosclerosis (AS), a pathological status of the vessels, is characterized by chronic inflammation, lipid accumulation, plaque formation, and lumen obstruction [1]. As atherosclerosis progresses, some plaques may become unstable, leading to plaque rupture, thrombus formation in the vessel lumen, and clinical events. Atherosclerosis and its complications significantly contribute to the morbidity and mortality of patients suffering from cardiovascular disease (CVD) [2].

The retention and oxidation of lipoproteins in the intima of arteries has been considered a fundamental event in atherosclerosis. Numerous studies have also shown that low-grade, chronic inflammation of the arterial wall is a component in the development of atherosclerosis [3]. This process could attract cells of the immune system into the atherosclerotic plaque, and the autoimmune response could switch from a protective to a pathogenic function as atherosclerosis progresses [4]. However, whether the switch in functionality represents a cause or consequence of atherosclerosis remains unknown [5]. At present, AS is not well understood in terms of its molecular and cellular mechanisms.

Pyroptosis is a type of programmed cell death that is induced predominantly by activating the GSDMD- (gasdermin D-) dependent or GSDME- (gasdermin E-) dependent signaling pathway in cells and exhibits morphological changes distinct from apoptosis and necrosis [6, 7]. Pyroptosis is an integral part of the innate immune system. Pattern recognition receptors (PRRs) activate pyroptosis when recognize pathogen-associated molecular patterns (PAMPs) and danger-associated molecular patterns (DAMPs) [8, 9]. PRRs are highly expressed in blood vessel cells such as endothelial cells and smooth muscle cells [10]. Once engaged, PRR signaling can activate pyroptosis and mediate the assembly of inflammasomes, leading to the production and release of cellular contents and inflammation [11]. Recent studies have provided evidence that pyroptosis initiates, deteriorates, and exacerbates atherosclerosis [12–14]. Zhang et al. reported that melatonin inhibits pyroptosis in endothelial cells via the MEG3/miR-223/NLRP3 axis and subsequently affects atherosclerosis [15]. The study by Meng et al. found that estrogen can prevent atherosclerosis by lowering the inflammatory response and pyroptosis activity in vascular endothelial cells [16]. Chen et al. also reported that the shear stress of blood flow at the arterial wall might cause the pyroptosis of vascular endothelial cells via the TET2/SDHB/ROS axis, offering new insights into the etiology of AS [17]. In addition, recent studies in the literature on the specific interaction between pyroptosis and adaptive immunity also indicated that pyroptosis might play a crucial part in AS [11].

Pyroptosis can be both the cause and the consequence of inflammation, and the respective roles in AS are difficult to decipher [18]. Although some regulatory factors and cellular processes acting on pyroptosis have been uncovered, technical limitations have made it impossible to fully explain pyroptosis mechanisms in AS.

The continuous development of microarrays and RNA sequencing technologies has become an effective tool for basic transcriptomics experiments and provides new insights into understanding the biology of atherosclerosis [19]. Therefore, based on bioinformatics analysis, the present study sought to identify pyroptosis-related gene expression patterns as well as validate the diagnostic value of those in AS.

## 2. Method

### 2.1. Data Collection

**Figure 1:**
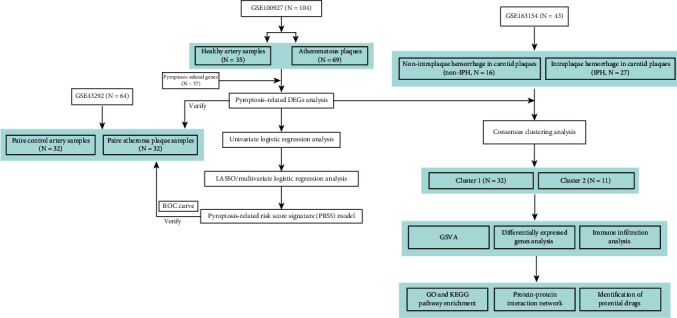
Flowchart of this study. DEGs: differentially expressed genes; LASSO: least absolute shrinkage and selection operator; GSVA: gene set variation analysis; KEGG: Kyoto Encyclopedia of Genes and Genomes; GO: Gene Ontology; ROC: receiver-operating characteristic.

RNA expression data were collected from three microarray expression datasets (GSE100927, GSE43292, and GSE163154) obtained from the Gene Expression Omnibus (GEO) database, which included atherosclerosis and control conditions [20]. The GSE100927 dataset includes 69 atherosclerotic plaques and 35 healthy arteries, obtained from the carotid, femoral, and infrapopliteal arteries. This dataset was performed using the GPL17077 platform [21]. Sixty-four samples from 31 patients with carotid endarterectomy were included in GSE43292. Atheroma plaque with the core and shoulders of the plaque was paired with macroscopically intact tissue from a distant location in the same patient. The expression profile arrays of GSE43292 were generated using GPL6244 platform [22]. In addition, the GSE163154 dataset consists of 43 pathologically detected absent (*N* = 16) or present (*N* = 27) intraplaque hemorrhages in carotid plaques. The expression profile arrays of GSE163154 were generated using the GPL6104 platform [23].  Figure 1 shows the flowchart of this study.

### 2.2. Identification of Differentially Expressed Pyroptosis-Related Genes

The pyroptosis-related genes have been identified through previous studies [24, 25] and the Molecular Signatures Database (version 7.5.1), which were listed in Supplementary Table 1. The differentially expressed pyroptosis-related genes between atherosclerotic plaques and normal arteries in GSE100927 and GSE43292 were screened using the “limma” package. The expression levels of pyroptosis-related genes were visualized using heatmaps and boxplots constructed with the “ggplot2” package. In terms of pyroptosis-related gene expression levels, principal component analysis (PCA) was employed to analyze dominant patterns and sample distribution.

### 2.3. Immune Profile Analysis

Immunedeconv integrates six algorithms to manage various cell component analyses [26]. The “immunedeconv” package (version 2.0.4) was used to analyze immune cell component analysis in different groups using the EPIC method. By using the “GSVA” package, we compared enrichment scores for multiple immune-related pathways gene sets between groups by using single-sample gene set enrichment analysis (ssGSEA) [27].

### 2.4. Development and Validation of a Predictive Pyroptosis-Related Gene Signature

To investigate associated variables in GSE100927, the expression of pyroptosis-related genes between atherosclerotic plaques and control arteries was compared using univariate analysis. LASSO regression was utilized to determine significant independent pyroptosis-related genes associated with AS [28]. Finally, using multivariate logistic regression, a pyroptosis-related risk score signature (PRSS) was constructed, and the formula is as follows: *P* = 1/[1 + exp(−*xβ*)] [29]. To obtain the logistic LASSO estimator, we used the “glmnet” package in R. The receiver operation characteristic (ROC) curve was performed, and area under the curve (AUC) was generated utilizing the “pROC” package to verify the diagnostic performance of the PRSS in GSE43292.

### 2.5. Consensus Clustering

The expression patterns of pyroptosis-related genes were used to categorize atherosclerotic plaques into various groups using an unsupervised clustering algorithm [30]. The consensus clustering approach was performed, and the “ConsensuClusterPlus” package was used to determine the number of clusters.

### 2.6. Gene Set Variation Analysis (GSVA)

GSE163154 was subjected to a genetic variation analysis (GSVA) enrichment analysis to determine the signal pathway for each AS sample by using the “GSVA” package. The KEGG pathway retrieved from the MSigDB database was used as the background pathway database. A statistically significant difference between the different clusters was defined as adjusted *P* < 0.05.

### 2.7. Analysis of Functional Enrichment of DEGs between Different Clusters

The DEGs between the two different clusters in GSE163154 were identified using the criterion: |log2FC| > 1 and adjusted P < 0.05. GO and KEGG pathway enrichment analyses for DEGs between different clusters were performed using the “ClusterProfiler” package (version 3.12.0). The protein-protein interaction (PPI) networks of DEGs were generated by the Search Tool for the Retrieval of Interacting Genes/Proteins (STRING) database (http://string-db.org/), and Cytoscape (http://cytoscape.org) was then used to visualize the PPI networks [31]. The top 20 genes with high node gene degrees were identified as hub genes, which were determined using the CytoHubba utilizing the maximum neighborhood component (MNC) method.

### 2.8. Identification of the Potential Drugs

The Drug Gene Interaction Database (DGIdb) (version 4.2.0, https://www.dgidb.org) [32], an online resource that provides linkages between genes and their known or potential drug associations, was used to identify potential medications and chemical substances that interacted with hub genes, and alluvial diagram was used to display the drug-gene interaction network.

### 2.9. Statistical Analysis

To compare differential expression of pyroptosis-related genes between two groups, Student's *t*-tests were used, and *P* value of 0.05 considered statistically significant. To investigate the discriminatory performance of PRSS in validation data, a receiver-operating characteristic (ROC) curve was constructed and the area under the ROC curve (AUC) was calculated with 95 percent confidence intervals (CIs). A model for prediction of atherosclerosis was developed using multivariate logistic regression. The statistical analyses were conducted with R (version 4.0.0; https://cran.r-project.org/src/base/R-4/).

## 3. Result

### 3.1. Expression Variation of Pyroptosis-Related Genes in Atherosclerosis

**Figure 2:**
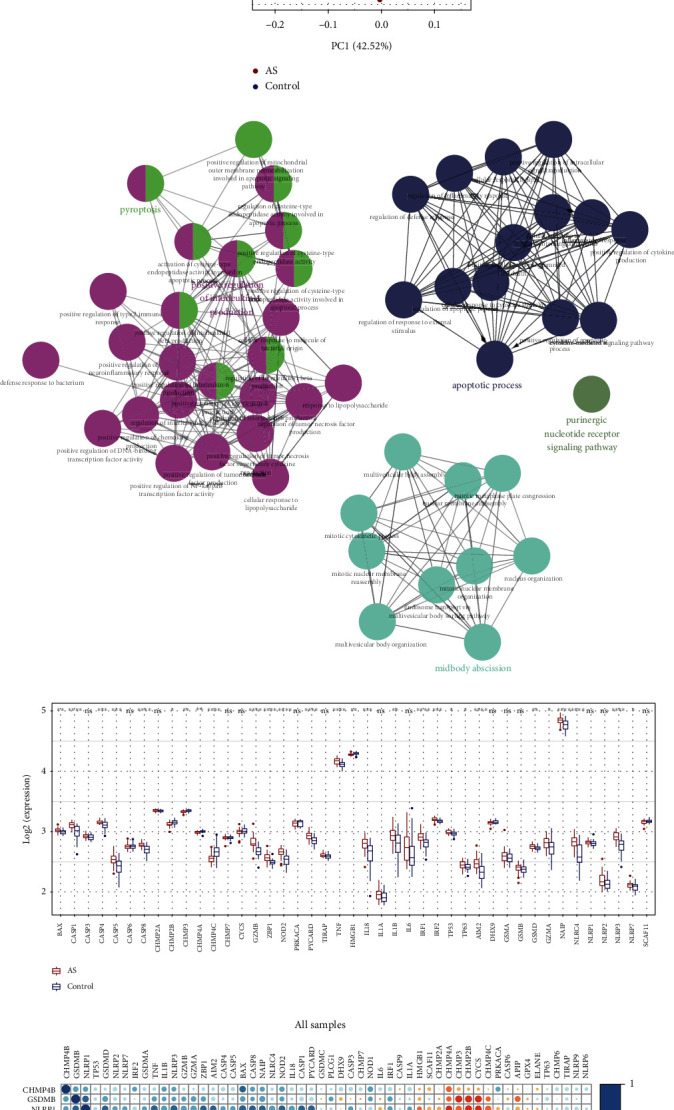
The expression of pyroptosis-related genes in atherosclerotic lesions. (a) Heatmap and (b) boxplot shown the expression of pyroptosis-related genes between normal and atherosclerotic arteries in GSE100927. (c) Principal component analysis (PCA) plot for atherosclerotic and normal arteries based on pyroptosis-related genes. (d) Gene Ontology (GO) enrichment analyses for pyroptosis-related genes. (e) Differential expression of pyroptosis-related genes between atherosclerotic arteries and their adjacent normal arteries in GSE43292. Analysis of pyroptosis-related genes expression correlations of (f) all samples and (g) atherosclerotic samples in GSE100927. AS: atherosclerosis. ^∗^*P* < 0.05; ^∗∗^*P* < 0.01; ^∗∗∗^*P* < 0.001; ^∗∗∗∗^*P* < 0.0001; ns: not statistically significant.

54 pyroptosis-related genes were identified in GSE100927, and the expression differences between atherosclerotic lesions (*n* = 69) and normal arteries (*n* = 35) were calculated. Figures 2(a) and 2(b) show 28 genes upregulated and 15 genes downregulated in atherosclerotic lesions. PCA results showed that atherosclerosis and normal arteries were better distinguished by pyroptosis-related genes (Figure 2(c)). Furthermore, enrichment analysis indicated that these genes were involved in pyroptosis and inflammatory responses (Figure 2(d)). To confirm the result, the expression of pyroptosis-related genes was tested in GSE43292. As shown in Figure 2(e), most pyroptosis-related genes are expressed differently in atheroma plaques and control samples. We also analyzed the expression correlation between the pyroptosis-related genes in all samples or only in AS (Figures 2(f) and 2(g)).

### 3.2. Construction of the Pyroptosis-Related Risk Score Signature

**Figure 3:**
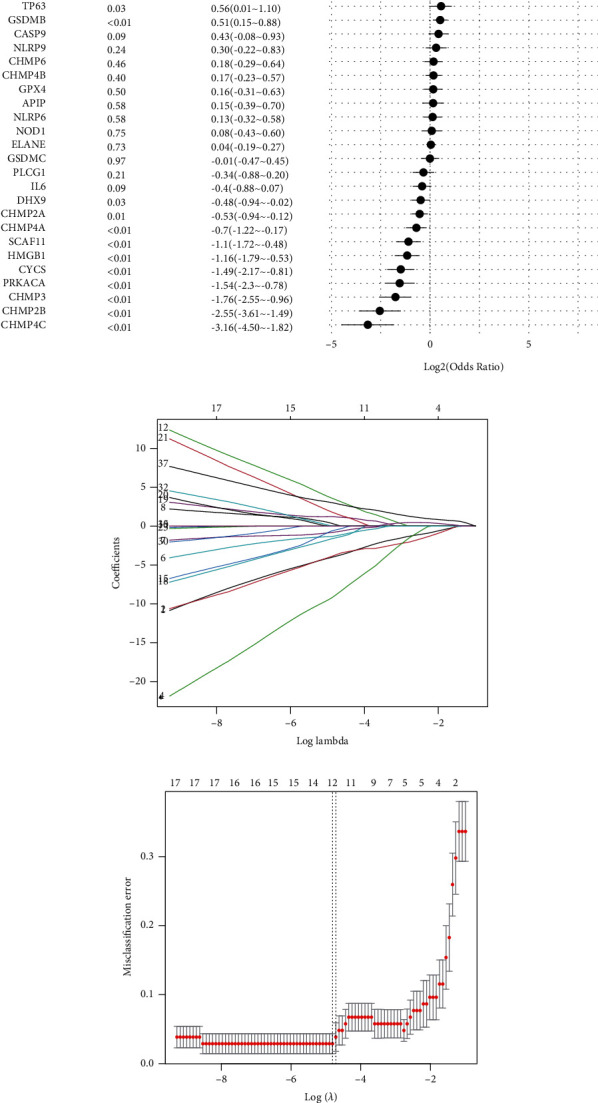
Development and validation of pyroptosis-related risk score signature. (a) Forest plot presenting the result of univariate logistic regression of pyroptosis-related genes. (b) LASSO coefficient profiles. (c) Cross-validation for tuning parameter selection in the LASSO model. (d) ROC analysis of PRSS for diagnosis of AS in GSE43292. PRSS: pyroptosis-related risk score signature; LASSO: least absolute shrinkage and selection operator; ROC: receiver-operating characteristic.

To investigate whether pyroptosis-related genes can be utilized as diagnostic biomarkers for atherosclerosis, in order to reduce the risk of overfitting, we used univariate logistic regression analysis (Figure 3(a)) followed by LASSO regression analysis (Figures 3(b) and 3(c)), and 12 genes were included based on the optimum *λ* value. Finally, the diagnostic pyroptosis-related risk score signature (PRSS) was generated using multivariate logistic regression, and 12 genes were able to enter the equation as diagnostic biomarkers, namely, CHMP2B, PRKACA, CASP5, CHMP4, HMGB1, CASP4, SCAF11, CASP6, IL1A, TNF, CHMP4A, and TP53. In GSE43292, the validation of the diagnostic efficiency of PRSS achieved a good level of performance (AUC = 0.816) (Figure 3(d)).

### 3.3. Atherosclerotic Plaque Classification Based on the Pyroptosis-Related Genes

**Figure 4:**
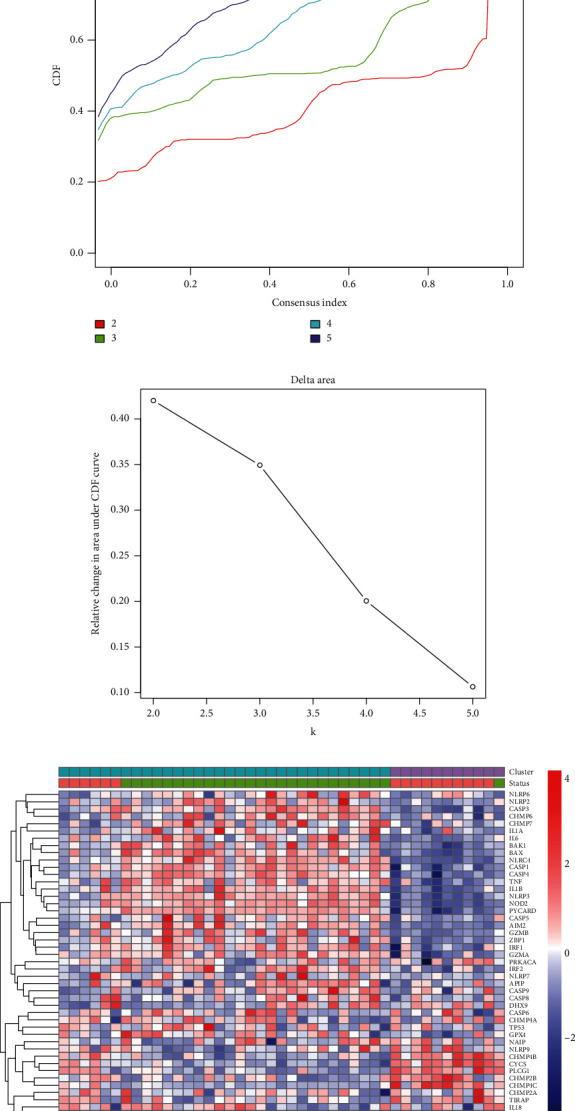
Pyroptosis subtype clustering in atherosclerosis. (a) Consensus matrices of the GSE163154 cohort for *k* = 2. (b) CDF curve. (c) Relative change in area under the CDF curve. (d) The clustering of pyroptosis-related genes among two clusters in GSE163154. (e) The heatmap for the biological pathways in different pyroptosis-related clusters. IPH: intraplaque hemorrhage; non-IPH: nonintraplaque hemorrhage; CDF: cumulative distribution function.

Consensus clustering was employed to determine the expression pattern of pyroptosis-related genes in different status of atherosclerotic plaques in GSE163154. To divide the samples in GSE163154 into different clusters, the clustering variable (*k*) was raised from 2 to 5. For the intragroup correlations were low, *k* = 2 was used to divide the samples into two clusters (Figures 4(a)–4(c)). As shown in Figure 4(d), most AS samples in cluster 1 were intraplaque hemorrhage (IPH) atherosclerotic (26/32, 81%), and the majority of samples in cluster 2 were nonintraplaque hemorrhage (non-IPH) atherosclerotic (10/11, 91%).

The biological behaviors between the two different pyroptosis-related clusters were explored by GSVA enrichment analysis. As shown in Figure 4(e), immunity activation pathways such as the RIG-like receptor signaling pathway and Toll-like receptor signaling pathway were enriched in cluster 1.

### 3.4. Immunological Activation in Various Groups

**Figure 5:**
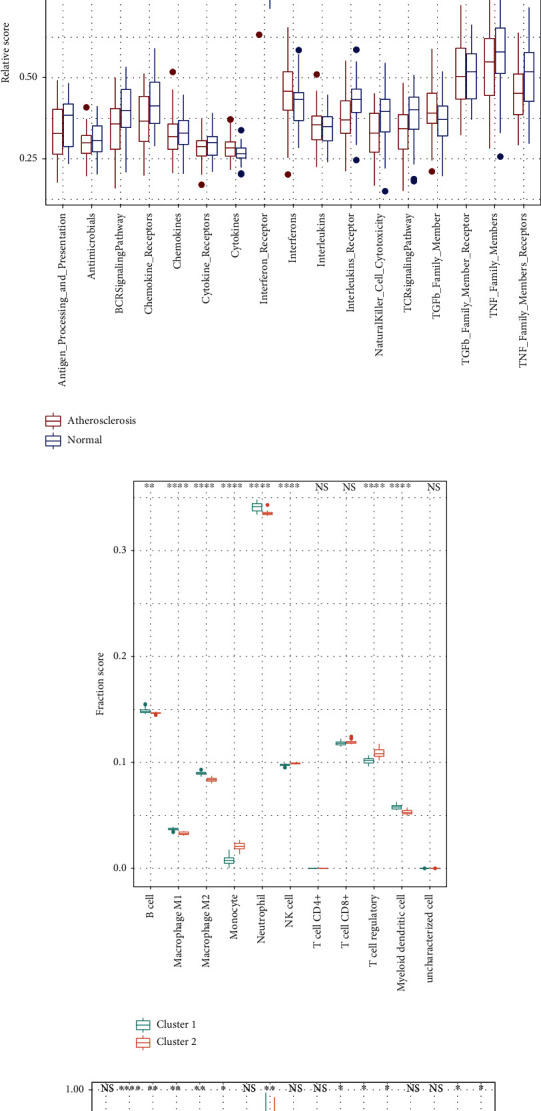
Immune status in various groups in GSE100927 and GSE163154. Boxplots for the (a, c) fraction scores of immune cells and the (b, d) relative scores of immune-related pathway gene sets. ^∗^*P* < 0.05; ^∗∗^*P* < 0.01; ^∗∗∗^*P* < 0.001; ^∗∗∗∗^*P* < 0.0001; NS: not statistically significant.

Based on the enrichment analyses, we used the immunedeconv and GSVA to analyze immune cell components and the immune-related pathway gene sets in different groups. In GSE100927, atherosclerotic lesions exhibited higher levels of B cells, neutrophils, macrophages, NK cells, and CD8+ T cells than normal arteries (Figure 5(a)). Except for the cytokines, interferon receptor, and TGF-*β* member receptor, the other immune-related pathway gene sets showed more activity in normal arteries (Figure 5(b)).

The relative proportions of immune cells differed markedly in the two clusters in GSE163154 (Figure 5(c)). Of these immune cells, NK cells, neutrophils, and T cell regulatory appeared in a higher proportion in the cluster 2 group than in cluster 1. The other five sorts of immune cells, on the other hand, had the inverse result. And the immune-related pathway gene sets showed more activity in cluster 2 than in cluster 1 during AS (Figure 5(d)).

### 3.5. Functional Analyses Based on the Pyroptosis Subtypes

**Figure 6:**
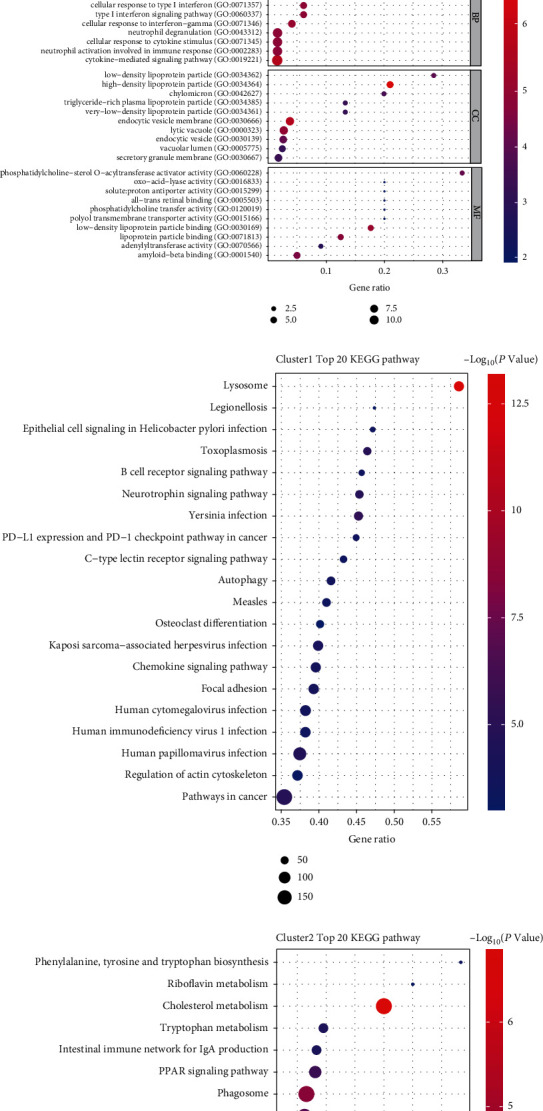
Enrichment analysis based on the DEGs between the two clusters. (a, b) GO and (c, d) KEGG enrichment analyses for the two pyroptosis-related clusters, respectively. KEGG: Kyoto Encyclopedia of Genes and Genomes; GO: Gene Ontology; BP: biological process; CC: cellular components; MF: molecular function.

In addition, the differences in gene functions and pathways involved in the two pyroptosis-related clusters were further investigated. Adjusted P < 0.05 and |log2FC| ≥ 1 were utilized as criteria for the extraction of DEGs by “limma” R package. Between the two clusters of GSE163154, 5475 DEGs were identified. 5426 genes were upregulated in cluster 1, while the other 49 genes were upregulated in cluster 2. GO enrichment analysis (Figures 6(a) and 6(b)) and KEGG pathway analysis (Figures 6(c) and 6(d)) were then performed in both clusters, and DEGs were significantly enriched in the immune response. In addition, the common biological processes in both clusters were neutrophil activation and degranulation.

### 3.6. Identification of the Potential Drugs

**Figure 7:**
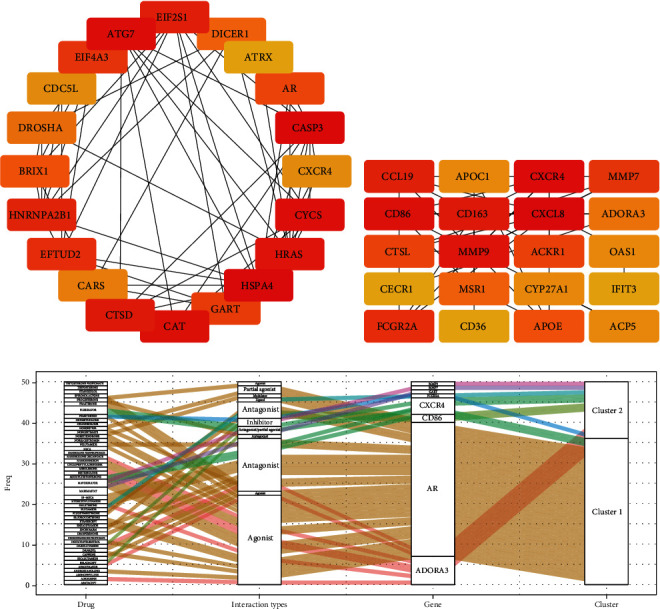
The drug-gene interaction network. Identification of twenty hub genes for (a) cluster 1 and (b) cluster 2. (c) Alluvial diagram showing the drug-gene interaction network. AR: androgen receptor; GART: phosphoribosylglycinamide formyltransferase; CXCR4: C-X-C Motif Chemokine Receptor 4; ADORA3: adenosine A3 receptor; MMP9: matrix metallopeptidase 9; FCGR2A: Fc gamma receptor IIa; MMP7: matrix metallopeptidase 7.

The PPI networks of DEGs were constructed (Supplementary Figure 1), and Cytoscape was also used to identify 20 hub genes in cluster 1 and cluster 2 (Figures 7(a) and 7(b)). The application of DGIdb is to select drugs or molecular compounds that have the potential to affect hub genes in two pyroptosis-related clusters. As illustrated in drug-gene interaction network (Figure 7(c)), we identified forty-seven molecular compounds or drugs. Prasterone, enzalutamide, and dromostanolone propionate were identified to differentially modulate androgen receptor expression (AR). In addition, pemetrexed has been found to interact with GART. Seven molecular compounds or medications, such as adenosine and aminophylline, have been associated with Adenosine A3 Receptor (ADORA3). Four molecular compounds or drugs modulated C-X-C Motif Chemokine Receptor 4 (CXCR4), while abatacept and belatacept modulated CD86.

## 4. Discussion

The most of the pyroptosis-related genes were found to be differently expressed between atherosclerotic lesions and normal arteries in this study. To further evaluate the pyroptosis-related gene signatures and the diagnostic value associated with AS, we constructed the PRSS model, which performed well in the external validation dataset. The current study also discovered that the expression patterns of pyroptosis-related genes differed significantly at two different statuses of atherosclerotic plaques. According to GSVA enrichment analysis, the two different clusters were associated with different metabolism and immune-related pathways. In both clusters, DEGs were associated with immune pathways as determined by GO and KEGG analyses. The activity of immune-related pathway gene sets, as well as the infiltration of immune cells in the two clusters, was investigated. The findings revealed a significant difference in immune response activation between cluster 1 (most cases were IPH) and cluster 2 (most cases were non-IPH). In addition, we used the DGIdb to identify molecular compounds and drugs with potential effects on hub gene expression regulation in atherosclerotic plaques.

We identified twelve pyroptosis-related DEGs from the GEO dataset as potential biomarkers for the diagnosis of AS. Among the pyroptosis-related genes, tumor suppressor p53 (TP53) has been identified as a classical tumor suppressor, which is also involved in lipid metabolism and atherosclerosis process [33, 34]. In the study by Kolovou et al., TP53 levels were upregulated in atherosclerotic coronary artery tissue, suggesting that the TP53 gene contributes to the progression of atherosclerosis development [35]. Consistent with that, this study found that TP53 was significantly increased in atherosclerotic plaques compared with normal controls. However, Wu et al. found that the absence of p53 could accelerate the development of atherosclerosis in ApoE^−/−^ mice [36]. CHMP4A belongs to the family of chromatin-modifying proteins and charged multivesicular body proteins (CHMPs). As a component of the ESCRT-III complex (intranuclear endosomal sorting complex required for transport III), it regulates cell cycle progression [37]. Previous studies have shown that after inflammasome activation, pyroptosis and interleukin-1*β* release could be greatly enhanced by inhibition of the ESCRT-III machinery in both human and murine cells [38]. In the present study, we found significant elevations of tumor necrosis factor (TNF) and interleukin- (IL-) 1 in atherosclerotic plaques. TNF encodes a multifunctional proinflammatory cytokine that plays a key role in mediating the inflammatory response in atherosclerosis, including induction of the expression of various cell adhesion molecules, monocyte/macrophage migration, and local proliferation [39]. The role of TNF-*α* in atherosclerosis has been extensively investigated [40]. Recent studies have shown that caspase-6 is involved in mediating innate immunity and promotes pyroptosis by facilitating the assembly of the inflammasome [41]. Although this study found that caspase-6 expression was significantly increased in atherosclerotic plaques, its role in AS is yet unknown.

In this study, pyroptosis-related gene expression was utilized to divide AS patients into two clusters, with cluster 1 being much more likely than cluster 2 to have intraplaque hemorrhage, suggesting that pyroptosis may affect the stability of atherosclerotic plaques. We further investigated the immune cell infiltration in different pyroptosis-related subsets. We discovered that B cells, macrophage M1, macrophage M2, and NK cells were highly expressed in cluster 1 compared with cluster 2, whereas Tregs and monocytes had low expression in cluster 1. The differences in immune cell expression were also found between atherosclerotic lesions and normal arteries. Macrophages have been found to play a key role in the formation of inflammatory response and the progression of atherosclerosis in previous studies [42]. It should be noted that atherosclerosis is a complex process with a dynamically changed microenvironment in which different subsets of macrophages may belong to pro- or anti-inflammatory types [43]. Tregs are known to protect against atherosclerosis and to be involved in different stages of atherosclerotic progression by suppressing inflammation [44, 45]. The decrease in Tregs during the progression of atherosclerosis was confirmed by single-cell RNA sequencing [46]. Classification of atherosclerotic plaques based on pyroptosis-related genes might help better understanding of the molecular processes of intraplaque hemorrhage.

According to recent research, pyroptosis has an impact on the entire course of atherosclerosis [47]. Therefore, appropriate intervention in the activation of pyroptosis in atherosclerotic plaques may provide a new therapeutic strategy for patients with AS [48]. To predict effective therapeutic agents for atherosclerotic plaques, the DGIdb was used to identify drugs and molecular compounds that affect upregulated hub genes in the two pyroptosis-related clusters. According to previous studies, the androgen receptor (AR) and androgens each play distinct roles in the atherosclerotic process. In alleviating atherosclerosis, targeting AR may yield better results than interfering with androgen expression because androgen deficiency leads to an elevated lipid profile [49]. ADORA3 encodes a protein that belongs to the adenosine receptor family and is part of the adenosine-mediated anti-inflammatory pathway [50]. The study by Yoshino et al. found that genetic variations within ADORA3 can mediate testosterone or its receptors, which has been associated with epicardial coronary endothelial dysfunction and has negative effects on cardiovascular morbidity and mortality [51]. A previous study has shown that selective enhancement of CXCR4 can maintain arterial integrity, endothelial barrier function, which could contribute to novel atherosclerosis treatments [52].

In this study, several limitations should be addressed. First, there were no clinical data from AS patients in GSE100927, the correlations between pyroptosis-related gene signature were investigated in datasets, and the individual characteristics of patients could not be analyzed. A prospective cohort of patients with atherosclerosis will be used to confirm the predictive efficacy of pyroptosis-related genes. Second, the atherosclerosis plaques in GSE100927 originated from different arteries. The process of atherosclerosis has a similar phenotypic pattern, even in different anatomical sites of the cardiovascular system [53]. Therefore, the pyroptosis-related genes derived from the deterioration of atherosclerosis at different anatomical sites might share some common characteristics [54]. Third, the accuracy of the discovery was limited because of the relatively small sample sizes of the study cohorts. Finally, no cell or animal experiments were performed in this study, so further experimental studies will be needed to verify our findings.

In conclusion, our study showed that pyroptosis activation was closely associated with the progression of atherosclerosis. Pyroptosis-related genes might be used as atherosclerosis diagnostic biomarkers. The expression patterns of pyroptosis-related genes in different statuses of atherosclerotic plaques may provide additional information about the deterioration of atherosclerotic plaques and guide therapeutic strategies.

## Data Availability

The data for this article is available on the NCBI-GEO website (https://www.ncbi.nlm.nih.gov/geo/), and further inquiries should be referred to the authors.

## Supplementary Materials

Supplementary Materials Supplementary Table 1: pyroptosis-related genes identified from previous studies and the Molecular Signatures Database; Supplementary Figure 1: construction of PPI networks by Cytoscape based on the DEGs in different clusters.
