# Supplementary material for: Pyroptosis-Related Gene Signature and Expression Patterns in the Deterioration of Atherosclerosis
Source: Dis Markers. 2022 May 5;2022:1356618. doi: 10.1155/2022/1356618 (PMC9098329; doi:10.1155/2022/1356618)
Supplement: Supplementary Materials — Supplementary Table 1: pyroptosis-related genes identified from previous studies and the Molecular Signatures Database; Supplementary Figure 1: construction of PPI networks by Cytoscape based on the DEGs in different clusters. [file 1356618.f1.zip › Supplementary Figure 1.docx]

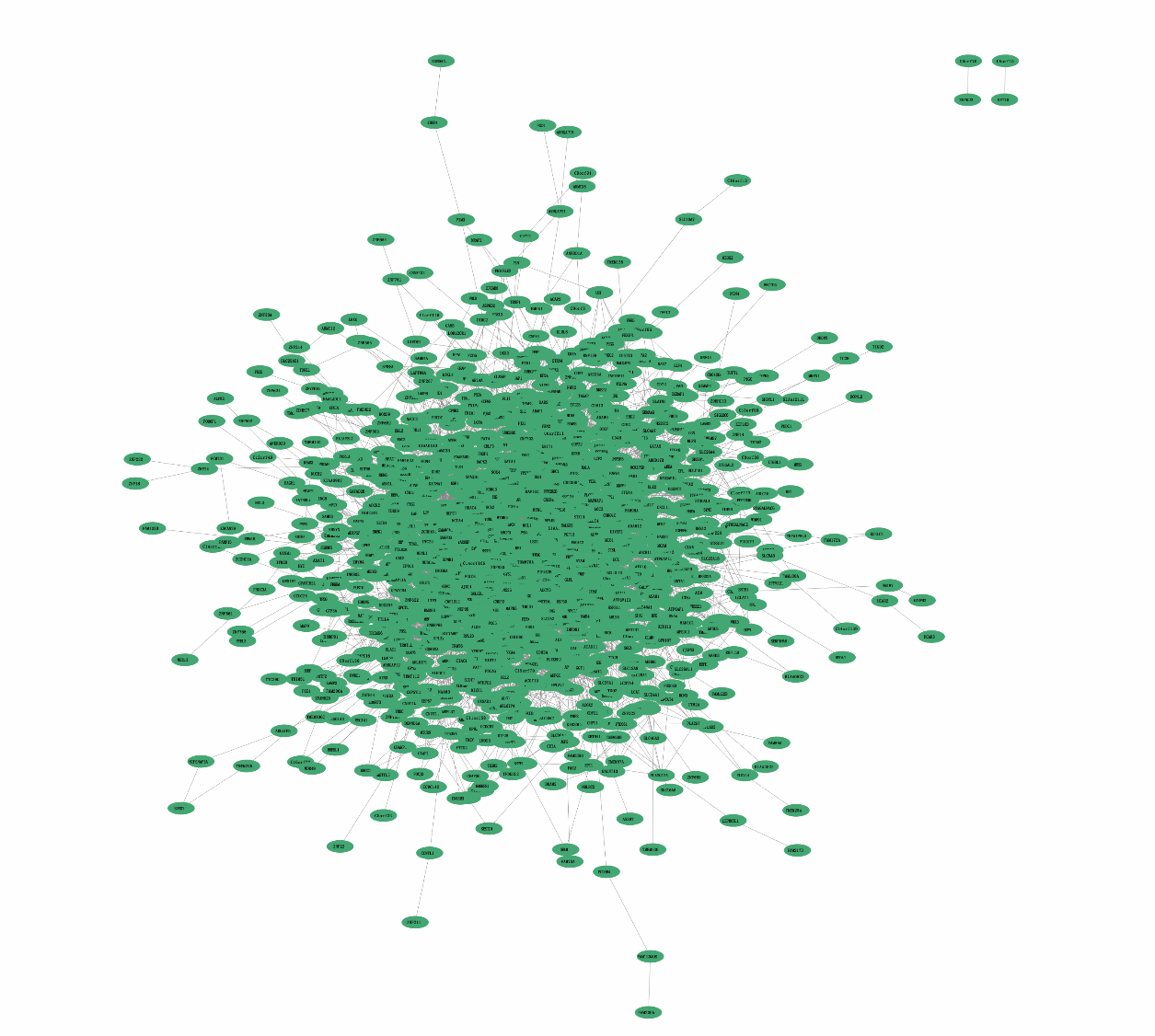


Supplementary Figure 1a: Construction of PPI networks by Cytoscape based on the DEGs in cluster 1


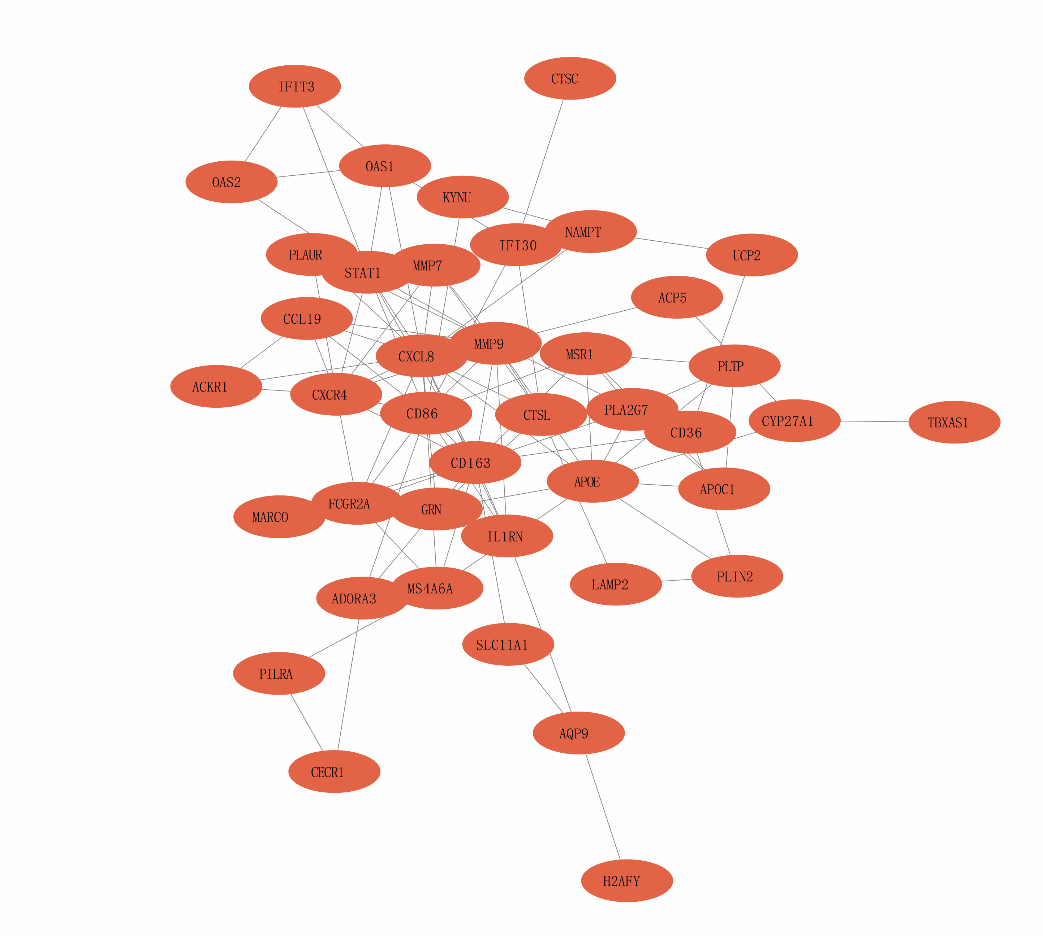


Supplementary Figure 1b: Construction of PPI networks by Cytoscape based on the DEGs in cluster 2
